# Supplementary material for: Comments by Microbiologists for Interpreting Antimicrobial Susceptibility Testing and Improving the Appropriateness of Antibiotic Therapy in Community-Acquired Urinary Tract Infections: A Randomized Double-Blind Digital Case-Vignette Controlled Superiority Trial
Source: Antibiotics (Basel). 2023 Aug 2;12(8):1272. doi: 10.3390/antibiotics12081272 (PMC10451981; doi:10.3390/antibiotics12081272)
Supplement: Supplementary file 1 [file antibiotics-12-01272-s001.zip › antibiotics-2487340-supplementary.pdf]

Supplementary Table S1. Comments displayed to General Practitioners randomized in the intervention arm, by case-vignette.

**Case vignette**	**Number 1**	**Number 2**	**Number 3**	**Number 4**	**Number 5**	**Number 6**
**Clinical situation**	Complicated cystitis	Male UTI	Uncomplicated pyelonephritis	Pyelonephritis at risk of complication	Bacteriuria in pregnancy	Indwelling urinary catheter-associated bacteriuria
**Sex**	Female	Male	Female	Female	Female	Female
**Age**	76 years of age	60 years of age	33 years of age	77 years of age	29 years of age	43 years of age
**Prior exposure to antibiotics**	No	No	No	Levofloxacin 4 months ago	No	No
**Pathogen**	P. mirabilis $>10^5$	E. coli $>10^6$	E. coli $>10^5$	E. coli $>10^6$	E. coli $>10^6$	E. cloacae $>10^5$
**Antibiotic susceptibility**						
Penicillins, Carbapenems, Monobactam						
Pivmecillinam	Sensitive	Resistant	Sensitive	Sensitive	Sensitive	Not tested
Ampicillin	Resistant	Resistant	Resistant	Resistant	Sensitive	Not tested
Amoxicillin-Clavulanate (cystitis)	Sensitive	Resistant	Sensitive	Resistant	Sensitive	Resistant
Amoxicillin-Clavulanate (other)	Sensitive	Resistant	Sensitive	Resistant	Sensitive	Resistant
Piperacillin-Tazobactam	Sensitive	Sensitive	Sensitive	Sensitive	Sensitive	Resistant
Temocillin	Sensitive	Resistant	Sensitive	Sensitive	Sensitive	Resistant
Ticarcillin	Resistant	Resistant	Resistant	Resistant	Resistant	Resistant
Ertapenem	Sensitive	Sensitive	Sensitive	Sensitive	Sensitive	Sensitive
Cephalosporins						
Cefoxitin	Sensitive	Sensitive	Sensitive	Resistant	Sensitive	Resistant
Cefixime	Sensitive	Sensitive	Resistant	Resistant	Sensitive	Resistant
Ceftazidime	Sensitive	Sensitive	Intermediate	Intermediate	Sensitive	Resistant
Ceftriaxone	Sensitive	Sensitive	Resistant	Resistant	Sensitive	Resistant
Aminoglycosides						
Gentamicin	Sensitive	Sensitive	Sensitive	Sensitive	Sensitive	Sensitive
Amikacin	Sensitive	Sensitive	Sensitive	Sensitive	Sensitive	Sensitive
Quinolones, Fluoroquinolones						
Nalidixic acid	Intermediate	Sensitive	Sensitive	Sensitive	Sensitive	Sensitive
Ofloxacin	Sensitive	Sensitive	Sensitive	Sensitive	Sensitive	Sensitive
Levofloxacin	Sensitive	Sensitive	Sensitive	Sensitive	Sensitive	Sensitive
Others						
Sulfamethoxazole/trimethoprim	Resistant	Resistant	Sensitive	Sensitive	Sensitive	Sensitive
Nitrofurantoin (cystitis)	Sensitive	Sensitive	Sensitive	Sensitive	Sensitive	Sensitive
Fosfomycin (cystitis)	Sensitive	Sensitive	Sensitive	Sensitive	Sensitive	Sensitive
**Comment provided**	« The first-line recommended treatment is pivmecillinam or nitrofurantoin »	« The first-line recommended treatment is a fluoroquinolone »	« The first-line recommended treatment is amoxicillin-clavulanate; the 2nd line treatment is a fluoroquinolone or trimethoprim-sulfamethoxazole »	« The first-line recommended treatment is a fluoroquinolone or trimethoprim-sulfamethoxazole »	« Colonization to be treated. The first-line recommended treatment is amoxicillin; the 2nd line treatment is pivmecillinam or fosfomycin tromethamol »	« According to guidelines, a urinalysis is not indicated except in case of pregnancy or for planned urological surgery»
